# Gender as a historical kind: a tale of two genders?

**DOI:** 10.1007/s10539-018-9619-1

**Published:** 2018-05-10

**Authors:** Marion Godman

**Affiliations:** 1 Department of Political and Economic Studies, University of Helsinki, Unioninkatu 40 A, P.O. Box 24, 00014 Helsinki, Finland; 2 History and Philosophy of Science, Cambridge University, Cambridge, UK

**Keywords:** Gender, Historical kinds, Cultural evolution, Scaffolded reproduction, Adaptationism, Common ancestry

## Abstract

Is there anything that members of each binary category of gender have in common? Even many non-essentialists find the lack of unity within a gender worrying as it undermines the basis for a common political agenda for women. One promising proposal for achieving unity is by means of a shared historical lineage of *cultural reproduction* with past binary models of gender (e.g. Bach in Ethics 122:231–272, 2012). I demonstrate how such an account is likely to take on board different binary and also non-binary systems of gender. This implies that all individuals construed as members of the category, “women” are in fact *not* members of the same historical kind after all! I then consider different possible means of modifying the account but conclude negatively: the problem runs deeper than has been appreciated thus far.

## Introduction

Most of us live in a culture in which gender is understood as a binary matter: you are either a woman or a man; you are either a girl or a boy. Gender is also a rich category in which to belong or identify with. It cuts across a wide variety of dimensions: personal characteristics, clothing and bodily aesthetics, work and relationship roles are all marked in a gendered manner. What is gendered as belonging to men (or boys) and what is gendered as belonging to women (or girls) also vary considerably across both time and cultures. A promising way of explaining how gender can be variably expressed while members still belong to the same *Kind* is to take gender as a serial collective (Young 1994) or a historical kind (Bach 2012), such that individuals are connected through a chain of cultural reproduction. In this paper I will argue that such an account nevertheless has limited potential with respect to ascertaining a desired unity within a gender category.

I begin by setting out the important features of gender, and leave us with a problem of unity and representation within a gender category to be resolved (“Sex, gender and the representation problem” section). Next, I present a plausible way to address the problem where gender is a historical kind based on cultural reproduction (“Gender as a culturally reproduced historical kind” section). Yet, in the section that follows, I will go on to show that if the account is taken seriously, it becomes increasingly doubtful that it can satisfactorily provide the unity within a gender category that is typically desired (“Beyond binary systems of gender” section). I then review some possible ways of extending the explanatory scope of the historical account to address the problem (“Matching the intended scope of gender” section). Finally, I offer my conclusions (“Conclusion” section).

## Sex, gender and the representation problem

It is not just gender that is a tricky notion; as has become increasingly obvious, so is sex. The binary sex distinction has come under fire in recent decades, as several people have questioned whether there really are distinct biological (hormonal or genetic) causes of binary sex categories (see e.g. Fausto-Sterling 1992; Oudshoorn 1994; Richardson 2010). To begin this discussion of gender, however, I believe it is possible to sidestep the ontology of sex differences—including issues such as whether there is a real sex distinction arising from binary causes, or a distinction merely on the basis that sex emerges dimorphically^1^? Regardless of whether sex genuinely makes sense as a binary biological cause or category (genetic, hormonal or something else), it is *ubiquitously* coded into, or strongly associated with, two mutually exclusive *gender* categories: men (or boys) and women (or girls). It is of course highly likely that the *perception* of sex as a binary matter will remain relevant in terms of explaining why gender typically also comes out in a binary fashion (Wood and Eagly 2012; Fine 2017, see also “Gender as a culturally reproduced historical kind” section).^2^ In fact, Sandra Bem labels this process *gender polarization*: “the organizing of social life around the male–female distinction, the forging of a cultural connection between sex and virtually every other aspect of human experience” (1993, p. 192). This also seems to be the thought behind the popular construal of gender as the social interpretation of sex. Nevertheless, it should be borne in mind that this interpretation, assuming a close connection between sex and gender, is not automatic. Indeed, as I discuss in “Beyond binary systems of gender” section, cultural systems have actively endorsed three or more genders. By implication, at least in some gender systems it is acceptable for one’s gender to come apart from and not simply to follow from one’s perceived sex.

What holds across cultures is that gender is an extremely potent category in terms of structuring social life. As Bem and other social psychologists have thoroughly documented, we are so used to gender pronouns, stereotyping and so on, that we are almost blind to how pervasive it is (Liben and Bigler 2017). With so many properties being gendered, it is quite natural to think of gender as a *social kind*, providing some grounds for inductive generalizations and generics^3^ (Bach 2012, 2016; Mallon 2016). Note that this conception of social kinds is not necessarily essentialist. Many accounts of social kinds, follow John Stuart Mill’s notion of (real) Kinds for the inductive (including, social) sciences ([1886]^1974^). There multiple properties can be associated with a certain Kind even though none of them essentially belong to it (see e.g. Boyd 1991; Millikan 1999, Godman 2015). Still these non-essential properties belonging to a Kind matter for the (social) sciences as they have inductive potential—and are thereby also useful in interventions that affect social and political change.

One remaining problem with using gender categories in generalizations however is that it effectively disguises the fact that many of the statements are false for many, if not the majority of individuals concerned. In fact, variation within each gender category seems almost as compelling as the inductive potential of gender. This is true even for core characteristics of gender. Women serve as combat troops and game hunters in some non-industrial societies, for example, and in some hunter-gatherer societies fathers show intensive care for infants (Wood et al. 2002). There are also individuals within each culture who constitute exceptions to most predominant gender norms of that culture, such as the Queen of England (Mikkola 2009).^4^

If one purports to explain gender in terms of uniform social roles, such variation will clearly be problematic (see Bach 2012). But if it is not a uniform social role across time and space that link individuals of a gender, what does? The issue of unity and commonality within a gender might not have been of much concern among others than linguists and philosophers had it not also been associated with a broader political problem of gender. Theodore Bach (2012, p. 234) refers to this as *the representation problem*, a problem which many feminist philosophers, including Haslanger (2000) and Allison Stone (2004), have also emphasized. Here is Iris Marion Young’s influential formulation of the problem: “We seem to be able to talk about women as a collective in some sense, even though women’s experiences vary considerably by class, race, sexuality, age or society” (1994 p. 722). She goes on, “[but] without some clear sense in which ‘women’ is the name for a collective, there is nothing specific to feminist politics” (ibid. 714).

For these feminist authors, the issue of commonality and unity within gender is tied to the crucial political function that taking women as a unified and distinct collective has. Here is Young again: “The naming of women as a specific and distinct collective, moreover is a difficult achievement and one that gives feminism its specificity as a political movement” (ibid. 718). As she recognizes this function of the term is present even in the justification of concrete policies, such as affirmative action. Let us consider two prominent justifications of affirmative action and other proactive anti-discrimination policies toward women. One is about compensating for or remedying historical injustices in past generations of women, and the other, perhaps less controversial, one is the claim that women as a collective are *currently likely* to face structural discrimination. A background condition for both of these claims—pertaining to both historical and current structural injustices—is that there is something uniting a group or collective of (discriminated) members. In fact, even the claim that an *individual* is likely to face injustice is a likelihood that the individual faces *qua* being a woman. This all brings us back to the same question: what does belonging to a gender really mean?

## Gender as a culturally reproduced historical kind

My aim in this section is to offer a plausible account that defends gender as a culturally reproduced historical kind. This position has been set out in response to the representation problem in the work of Iris Marion Young on the one hand, who suggests that women should be thought of as a “serial collective” (1994), and in more detail in the historical essentialist account of gender defended by Theodore Bach. The latter is the account I will follow most closely (Bach 2012, 2016). I will try to demonstrate the virtues of the account, and in particular I will show that a historical account has the capacity to resolve any tension between the inductive potential of gender and the variation within the category.

If we want to consider a paradigm of historical kinds, species are often suggested (Millikan 1999, 2000; Bach 2012). But in my mind, there are far simpler cases of historical reproduction that can serve as a starting point. Take, for example, the true statements about Leo Tolstoy’s *War and Peace*: what makes them true? Here are some instances of *War and Peace*: the paperback on my bookshelf with the front page torn off, the one in my local library, the recent BBC series, and the superior Russian film version from 1969. All of these are members of the same historical kind as they are *all copies of an original version*. The fact that they are copies also *explains* certain reliable common features: the main protagonist of *War and Peace* is Pierre; the central love story in *War and Peace* is the one between Natasha and Prince André; *War and Peace* is set in Russia during the early nineteenth century.^5^ Perhaps this is a rather mundane set of claims. Scholars of literary and comparative studies may be far more interested in variations between instances and literary works. Yet, such studies arguably only make sense against a background of kind-identity between items. The account claims that such kind-identity is granted by the historical reproduction between items.

We thus already have an initial grasp of how inductive potential and intra-kind variation need not be in a state of mutual tension; indeed the latter seems to presuppose the former. The reader might already protest that a gender cannot be a kind in the same sense as *War and Peace* simply because it is much less evident that there is significant copying in the case of gender than in the case of *War and Peace* in which reproduction is obvious and the fidelity amongst instances can be assured. But the shared features of *War and Peace* hardly constitute a trivial outcome. After all, they demand, among other things, the invention and successful execution of the printing press, well-maintained technologies and legal institutions to ensure common features amongst instances. It is true, nevertheless, that there may not be a clear grasp of reproduction as a concept. At first pass, a historical kind seems to require the following main ingredients:

1. The existence of a model or models;
2. New member(s) produced in interaction with the model (or other past members);
3. The interaction with past models (or members) *causes* the new members to *resemble* past member(s);
4. Steps 2–3 *recur* such that there is chain of reproduction or *lineages*.

With regard to *models of gender* (1), Eagly and Wood (2005) suggest that it was the physical differences between the sexes—such as pregnancy and lactation among women and the greater size, speed and upper-body strength among men—that originally gave rise to the original gender differences. In their account, for example, this led men to perform tasks that required upper-body strength for extended periods of time, uninterrupted by having babies. It is quite plausible that real, or at the very least perceived, physical sex differences played an important role in the original emergence of gender models that were then transmitted through social learning. It is worth noting, however, that proponents of the historical-reproductive account of gender do not have to presume any particular account of *how* specific binary models (or further gender models) emerged alongside sex differences. Let us, again, consider *War and Peace*. The question of how or when Leo Tolstoy’s ideas and production of the work emerged, is distinct from the claim that subsequent instances are all copies of that original *however* the original emerged. On the other hand, whereas there is good reason to assume a *common cause* or *model* in the case of *War and Peace*, this is not at all obvious in the case of gender. In other words, given the likelihood that different cultural systems were spatially and temporally isolated from one another, current binary systems might, in fact, have *different* origins.

I will return to this question and its implications for solving the representation problem in the next section, but for now it suffices to note that there is no need to assume a particular account of the *emergence* of models of gender to make the case for a plausible reproductive link between members. For now, the question concerns how interaction between individuals could proceed such that new members come to resemble their predecessors.

The third condition of reproduction is that the interaction between past and present members has some modal force. In other words, it is precisely *because* the interaction produced similarity amongst members that new members were also produced. It is the nature of the interaction itself that makes it no accident that new members of a historical kind are produced. The copying of genetic material is one form of such reproductive interaction, but Peter Godfrey-Smith introduces what is perhaps a more common phenomenon: *scaffolded reproduction* (2009, Ch. 4 and 8). In scaffolded reproduction as opposed to, say, sexual reproduction, no material is exchanged and the reproduction relies heavily on the machinery outside of that which is reproduced. Scaffolded reproduction is the predominant model of cultural transmission, such as in the case of *War and Peace*. It also seems to be what happens in the case of gender.

Theodore Bach lists a number of causally interconnected components of a binary gender system that could be considered part of the machinery of the scaffolded reproduction of gender and gendered traits. These components include: binary sexual categories; conceptual gender dualism (including stereotypes, and psychological essentialist bias); gender norms (moral disapproval of individuals’ deviation from, and the moral approval of conformity to gender norms); gender roles and identity; binary gender-socialization practices; certain social and legal institutions; and binary gendered artefacts (2012, pp. 247–248). Amongst these elements, perhaps the most basic issue is *why* it is that individuals learn from certain models of gender and the horde of characteristics that belong to them.^6^ There are two plausible sets of reasons suggested in the literature: a sense of identification or belonging that *motivates one* to be of a gender; and the imposition via external socializing forces that *compels* individuals to become of a gender.

The first set of motivations are primarily studied in the literature on gender identity. In fact, Wood and Eagly (2015) make a useful distinction between two different traditions of gender-identity research: one that measures the degree to which an individual *identifies with gendered traits* (often coded in terms of masculinity and femininity) and one that measures the degree to which an individual *identifies as a member of a gendered group*. According to the former tradition, gender identity could be thought of as a judgement about, or recognition of, how similar one is to certain gendered stereotypes in terms of the traditional division of labor roles, for example. The latter tradition broadly aligns with the theory of social identity in social psychology (Tajfel 1981). Here the motivation for “self-categorizing” in terms of a gender is that it allows certain in-group ascriptions and at the same time allows distance from certain out-groups (Hogg and Turner 1985). Thus, motivations concerning in-group belonging, or simply arising from perceived similarity with others, offer one set of reasons why individuals pick the models that they do.^7^

Bach, however, believes that at heart gender is something imposed on the individual by external social pressures. This may happen through the desire to abide by (gender) norms to attain social approval, or indeed a fear of disapproval or punishment if one were *not* to conform to the norms of the perceived sex or gender (see also Castro et al. 2010).

Although it is common wisdom in cultural evolution theory that one learns more from some individuals than from others (e.g. Henrich and McElreath 2003; Lewens 2015), it is still worth asking why gender models are so much more powerful than other culturally available models of social learning (such as hair color and class). Perhaps part of the answer lies in the intensive and extensive role that gender plays in many areas of life that are likely to matter to the individual; or perhaps, as the latter cultural evolution tradition tends to stress, it lies in the *degree* to which society tends to reward conformation with one’s gender and to disapprove of gender or sex deviation.

In any case, one is now in a position to see how the account could resolve at least the initial puzzle. If a person’s gender is a result of accepted cultural reproduction from a chain of previous models, it can explain how the existence of binary gender roles can be reconciled, with each gender displaying both variation within cultures and change over time. Although each member of a gender is reproduced within a historical lineage that can be traced back to different models of gender, the lineage need not sustain all or even most properties originally associated with either of the binary models. At the same time, the binary lineages are maintained insofar as whenever each branch of gender undergoes change, a relevant binary contrast or “cultural dimorphism” is maintained between the two lineages. In other words, as long as neither gender becomes extinct (that is, the terms “women” or “man” refers to an empty set), significantly branches off from a lineage, or merges with the other, gender remains a binary matter and it is patterned in two distinct ways.

It might also seem that the unity of a gender simply follows from the shared reproductive history of each lineage. The idea of continuity amongst members is attained through a lineage of reproduction that has remained sufficiently “intact” over time and has avoided merging, extinction or considerable branching-off. But this conclusion is arrived at too hastily. The claim so far is only about gendered reproduction *regardless* of the number of original models. I have already flagged a worry that pervasive binary gender systems are not necessarily traceable back to common models (as in the case of *War and Peace*), now I turn to this worry more explicitly.

## Beyond binary systems of gender

If gender is a historical kind, then it should be clear that the current binary gender system need not necessarily have unfolded in a binary manner. In fact, I have shown just how contingent this system is on two features of reproduction: an existing cultural niche with binary models and the existence of gender-based social learning.

Indeed, given the view of gender as binary cultural lineages one can see that there are three different ways in which a culture can depart from having binary genders. First, the binary lineages might merge at some point, or one or both genders might cease to exist.^8^ Second, it may be that the original gender models were never binary at all (perhaps they were three or even more). Third, new alternative genders may emerge either because an existing lineage has locally branched off into one or more new gender lineages, or due to the *partial* merging of the two lineages into three.

Genderless cultural systems (option 1) seem as rare as those in which gendered social learning does not occur (perhaps for the same reasons).^9^ Although many *individuals* certainly identify in ways that cut across gender and perceived sex,^10^ it is hard to find *cultural systems* that have departed totally from gender-based social learning. The latter two options on the other hand—where categories of gender have always been, or have become, non-binary—have, in fact, featured in multiple cultural systems. One of the longest-lasting alternative ‘genders’ to achieve stability is the male ‘hijra’ in Pakistan and certain parts of India dating back to the sixteenth century (Nanda 1994). Another is the “berdache”, documented in 130 North American tribes with both males and females having assumed alternative gender roles (Roscoe 1991, p. 5). Anthropologist Will Roscoe documented the life in the late nineteenth century of a particular berdarche, We’wha, who lived among an isolated Zuni tribe near the south New Mexico–Arizona border. In response to the question of how one should make sense of the We’wha’s gender, Roscoe explicitly states that it is a matter of a third gender within the Zuni tribe:

The answer to the question “Was We’wha a man or a woman?” is “Neither”. That is, the sequence of initiations and social experiences that served to “cook” berdarches did not correspond to the sequence by which either men or women were “cooked”. We’wha represented a third possibility in the Zuni organization and representation of gender—a third gender status. (1991, p. 145)

I contend that these alternative or third genders should be considered historical kinds of gender in their own right. A new model (or in the case of more than three genders, several models) has been introduced and reproduced in accordance with the process of gendered social learning described in the last section. Roscoe suggests that in the case of the third-gender status (or *Ihamanaye*) of We’wha, there are two main arguments for stabilized reproduction. First, as noted in the above quotation, belonging to *any* gender in the Zuni tribe is part of a formalized process of cultural reproduction. In particular, it is a matter of ceremonial initiation rites in which a person passes from the genderless “uncooked” to the “cooked” stage of belonging to one of at least three genders, and becomes versed in accompanying learned practices such as dress and mastered craftsmanship—especially pottery for the Zuni berdarche (ibid. p. 123ff.). These practices serve to “scaffold” the reproduction between past models and new members of a particular gender. Second, the Zuni berdarche is an attractive model of social learning in that berdarches in the Zuni and in other North American tribes have occupied prestigious positions on many levels of their society—from being integrated into the origin myths to taking an active role in societal governance (ibid. p.147ff.). Under the hypothesis that third genders are historical kinds in their own right, these values and norms sustain the continued reproduction of the gender. Of course, once the cultural system perishes, so does the life-support system for such genders (as, sadly, is largely the case with the berdarches of the Zuni tribe).

What cultures in which gender is not strictly binary demonstrate, is not merely the contingency of the dominant binary cultural gender system, but also the likelihood that many cultural systems of gender do not share common origins or a common ancestry. It is far more likely that models of gender—binary and non-binary—have emerged separately in different cultures. If this is so, it does not matter how prevalent non-binary cultures are. What is more important, these differently arranged gender systems hint at a *separate ancestry (and ancestors)* for the terms “man” and “women” *in general*, which thus far have been thought of in terms of shared ancestry. If one wishes to tell a tale of historically reproduced gender, therefore, it is less likely to be a tale of two genders. It will rather be a tale of binary system*s* of gender (and some of which are not strictly binary) than of *a* binary system, each one containing *different* historical kinds of gender.

This could spell trouble for the unity of gender. If the assumption of common ancestry is an unlikely hypothesis, then there simply is not a historical chain uniting all individuals categorized as “women” and all those categorized as “men”. True, one could still say that each individual “woman” is modeled from *a* gender variant within *some* binary cultural system of gender but without common ancestry one is not entitled to assume that each of these individuals will share a unifying feature. In fact, the survey into non-binary systems also implies that such a proposal already excludes individuals who are gendered as part of *non*-*binary* systems. Nor can one claim to be concerned with one of the two genders in a binary system, or one of three genders in a non-binary system (and so on), since that presupposes an account of *which* of the two or three genders (and so on) one was talking about to begin with. Is there any way to salvage the notion of two genders with the broad scope that is typically intended?

## Matching the intended scope of gender

As noted above, Bach advocates a historical cultural-reproductive account of gender that aims to address the representation problem—as well as other problems. Accordingly, he thinks taking gender to be a historical kind gives a superior answer to accounts that treat it as an “objective type”, which makes a salient normative feature of gender, with which one may be concerned in political advocacy, essentially a matter of definition (2012, 2016).

[A]n ontology of historical kinds provides explanations for objective similarities. The present proposal agrees with Haslanger’s social objectivism that women generally share the objective similarity of social subordination. But rather than making this property definitive of women, it explains this property as one of several properties that reliably co-occur as a result of a more fundamental, historical property. (2012 p. 271)

First, allow me to state that I fully agree with Bach about not building in the normative feature that might concern us as a matter of definition of what it is to be of a gender—not least because *if* it is a matter of definition, we would not be able to have substantial disagreements about those gender claims. Therefore, contra Haslanger (2000, 2012), I do not believe that it serves political causes of emancipation to render “women are subordinated” and “men are dominant” as claims that are true nominally or by definition: they should be truth-evaluative.

However, as was made clear in the previous section, the view that gender is a historical kind comes at a cost. In particular, it does not satisfactorily resolve the representation problem that Bach aims to address, simply because there is no guarantee of tracing the common descent of all “women” and “men” to the same binary models and ancestors. As I have shown, this is not merely a theoretical possibility. It is rather grounded in evidence of the existence of multiple gender systems—some of which are non-binary—between which there has been no cultural transmission.

Although Bach does not seem to appreciate the extent of the representation problem for the account, he does offer what one might first see as a remedy: an appeal to *teleological functioning*. According to Bach, items that are replicated from a historical lineage due to selection have a teleological function (2012, p. 244 ff.) For example, pumping blood is a teleological function of the heart because its function is part of the reason that hearts are reproduced—it bestowed bearers of hearts with certain fitness advantages. He supposes that the same holds for a gender system: the advantage originally conferred by certain traits belonging to a gender system is also the reason why these traits are reproduced. It is the favorable effect of gendered traits within a cultural gender system in the past that causes descendants of that culture to continue to possess them.^11^

In Bach’s view this means that an individual may possess membership in a historical kind of gender as well as in a teleofunctional kind—the latter being a kind with an analogous cultural function to other members. Consequently, women with different historical models may still share membership in virtue of the teleological function. One can therefore speak of sameness in teleological functioning across different gender lineages as well as sameness in the *breakdown* of the function. Bach gives the following example:

American and Japanese women are not members of numerically the same historical kind. However, the historical gender roles in each system are analogous. On account of their shared type of history, then, American and Japanese women are members of numerically the same teleofunctional gender kind. This means that both American and Japanese women can fail to satisfy their teleofunctional gender norm and yet both are still members in the cross-cultural teleofunctional gender kind (and, of course, they are still also members in their respective historical kinds). (2012, p. 262)

Bach does not suggest it directly, but it may be possible to hold on to a tale of two genders if they are grounded in a shared teleological function rather than history. It is because of the shared function between numerically different historical kinds of gender that two teleofunctional gender kinds arise. For instance, common selection pressures may lead women to be barred from certain leadership roles in different gender lineages, which is then attributed to a common telefunctional role amongst all historical kinds of women. Or, common selection pressures may lead men to abuse their power for their sexual advantage in different lineages, and this explains a common telefunctional kind, men.

But what, then, is the real status of selection or selection pressure in these examples? If the teleological function is not to be merely a nominal status, there must be some way of knowing which features should form the basis of a teleofunctional kind.^12^ To continue with the same example, there must be some reason to think that it is indeed power imbalance that is the cross-cultural explanation for why traits such as submissiveness and abusiveness re-occur (also in different lineages). This is an empirical guess, which is in fact more difficult to support than it may seem. First it seems plausible that it would take a relatively long time for a gender kind to acquire a shared teleological function—even in a culture. It is known, however, that over large chunks of time and space, many cultural selective pressures on gender are bound to vary both within and between lineages. Let us revert to one of the examples: how do we ground the tendency to abuse power for sexual advantage amongst contemporary middle-class men in Finland and contemporary working-class men in the Philippines, as well as between male aristocrats in either of these locations 300 years ago? Although I am not denying that there may be such a common tendency amongst these individuals, common selection pressures amongst lineages does seem like a bit of a stretch. Why could it not be social learning in some cases, a by-product in others, and individual learning in yet others?

In truth, worrying about appealing to analogous functions is an instance of the more general worry about the tendency to prioritize adaptationist hypotheses in evolutionary theorizing and to dismiss alternative causal explanations as simply “null hypotheses” (Lloyd 2015). As Richard Lewontin and Stephen J. Gould drew our attention to, not all traits and products of biological evolution being due to selection; some arise via drift or by being “spandrels”—i.e. bi-products of other (cultural) adaptations (1979). In the case of cultural evolution there are even more alternatives to adaptationist hypotheses in that not only are there traits that are by-products of some gendered traits, some traits may develop as a result of individual trial-and-error learning.

I do believe that Bach is right in highlighting the importance of norms and beliefs about a hierarchy of social statuses, the division of labor, and sexuality in the reproduction of gender in distinct lineages. However, whether these beliefs and norms generate any meaningful analogies and teleological roles between lineages so as to constitute new gender kinds, is another matter. I would make a much more modest claim, namely, that social norms and beliefs play a part in *regimenting* the reproduction of gender in a binary manner in diverse lineages.

I therefore propose sticking to gender as historical kinds. In other words, any reference to a gender is always a reference to a particular historical kind (e.g. Japanese women, say) with common ancestry and the spatial temporal proximity of copying as guiding the right individuation of kinds. Of course, this still allows for cultural selection to be the most plausible empirical hypothesis in terms of trait retention within a particular lineage, but it is then precisely a matter of justifying a selected trait *within a lineage*. It is different identifying analogous traits *between* lineages that give rise to kinds in their own right.

Nevertheless, getting rid of teleofunctional gender kinds might seem to come at a significant price. As noted, historical membership alone seems disappointing if the aim is to unify the collective of women. Such disappointment might motivate the appeal of a shared adaptive role between different binary systems of gender: they are united by some overlapping cultural adaptive roles. Without a shared history or a shared adaptive function is there any hope of solving the representation problem? Iris Marion Young at least was clear that the representation problem is a difficult but nevertheless necessary problem for feminist political movements to confront. It seems as if there is no getting away from the fact that many of these movements aim for a conception of gender that transcends the particular cultural system in which gender is reproduced. Is there any way an account of gender as historically reproduced kinds could meet such demands? In my view, there are two options.

The first option is to extend the scope of gender by appealing to lateral transmission between different binary systems. After all, gender identification and imposition within a historical lineage may evoke a sense of solidarity, and eventually cultural transmission, that transcends the existing lineage. Let us say, for example, that injustices such as silencing and harassment take place within the lineage (A_1_) in one cultural niche (A), and that this fosters identification with a similarly unjustly treated gender from a different lineage (B_1_) within a different cultural niche (B).^13^ The likely result is that there will be cultural transmission and social learning between the different lineages A_1_ and B_1_—not least in how to develop strategies to deal with and overcome these injustices. The end result is thus that there is both some lateral transmission between lineages as well as some partial convergence in new models of social learning. Values of solidarity and concern could promote partial convergence of gender lineages by helping to generate new common models and hence common ancestry. In fact, the convergence of new models of gender is probably more likely given the prevalence of social media, technology and travel that harnesses existing tendencies of gendered social learning (and hence without needing the mediation of solidarity). Lateral cultural transmission is thus likely to extend the scope of membership in a historical kind of gender somewhat by merging different binary systems—particularly during the past couple of generations. Nevertheless, this option is still likely to exclude many gendered individuals from inclusion in common membership. In particular, there will be many historical cases in which such lateral transmission is impossible, even if individuals still belong to lineages within binary and non-binary gender systems. (For what it is worth, I think the common agency within these different gender systems might be better understood in terms of coalitions and alliances rather than being based on membership of a common kind.)

At this point the second option emerges. Why not interpret gender as historical kinds, but according to a biological sex, rather than to a culture?^14^ In that case a “gender” will encompass all humans with a particular biologically reproduced sex (or reproductive system)—supposedly male or female. This would involve taking a stand with respect to the existence of two biological sex categories, however. As mentioned at the beginning of this paper, such biological dimorphism requires independent argument, especially in view of there being plausible intersex categories. Moreover, such a switch seems to imply that sex and gender also map onto each other—a connection that, as noted, is tenuous to say the least, given the controversial taxonomy of sex and the existence of both non-binary and cross-genders. In particular, one may end up excluding individuals who are gendered in a different way from their (perceived) sex. Hence, I do not believe that replacing gender (as a historical kind) with sex (as a historical kind) is a winning strategy for extending the scope of gender, either.

## Conclusion

My current diagnosis is that the representation problem should not be fixed by sacrificing a plausible explanatory story of gender that takes gender to be a historical kind. After all, understanding gender as cultural reproduced kinds makes sense in terms of how variation over time within a lineage can be reconciled with the kind displaying an ample amount of culturally inherited attributes. It is important for the science of, and policy on, gender to respect both the inductive potential of as well as the variation within a gender category—even within a culture. Certainly sex alone cannot explain these features.

Moreover, an account stating that gender is a historical kind because of cultural reproduction makes sense of why claims about gender are typically more plausible if they are more narrowly indexed—or, in the terms of the account, indexed to specific lineages of reproduction. In other words, both political movement and social science should respect the particular cultural niche in which gender is reproduced and should be cautious in making sweeping cross-temporal and cross-cultural claims. It thus seems that we are at least *prima facie* correct in applying skepticism to many far-reaching prejudicial gender generalizations and generics, given that in many cases there may rightly be nothing uniting the group represented in the subject in the first place!^15^

Still. I have ended on a slightly negative note in terms of Young’s original hope of finding something on which to base the political movement of feminism. It turns out that the representation problem runs deeper than has been appreciated in discussions proposing historical kinds of gender. As it stands, the historical account may not give the full generality that was initially hoped for. This leaves us with two remaining options: reconsidering how desired a tale of two genders is, or thinking harder about an explanatory account of gender that will truly yield two timeless and cross-cultural genders.

## Footnotes

1 See a recent suggestion by Joel (2016).

2 This is not to ignore Judith Butler’s influential argument that we enforce categories and norms about sex precisely to underline our gender norms and categories (1999). It is merely to suggest that regardless of the legitimate reach of sex categories, the behaviour and dimensions of gender are broader.

3 *Generics* or unquantified generalizations is the typical linguistic tool for asserting properties that belong to a gender: “Women are caring”, “Typically men like to watch football” and “Generally dolls belong to girls” (Leslie 2008). However, I will mostly use *generalizations* in what follows denoting both the quantified and unquantified kind.

4 As one reviewer pointed out, however, arguably the most predominant gender norms of femininity concern appearance, in which case the Queen of England—God bless her matching hats and handbags—certainly conforms.

5 It may seem as if the *War and Peace* claims are about a single individual work rather than a Kind. Nevertheless, it is expected that what is true for the original version is also true for most of the copies, e.g. the book on my shelf. Indeed, it is for this reason that Millikan suggests individuals (including individual works) follow the same structure in underwriting generalizations (2000, Ch. 2).

6 That is not to deny that one value of gendered concepts and distinctions is purely *epistemic.* Gender concepts are reproduced to facilitate knowledge of the nature and distinctions between existing genders. In short, if one wants to be able to orient in a complex social world that has pre-existing gender structures, gender proves to be a useful heuristic. If people (and other things!) are already gendered in two distinct ways in one’s culture, it is only natural that learning about and navigating gender is an *epistemic priority*.

7 I write gender *or* sex as we are often we are not aware of a distinction and in many cases it does not matter, because even just a perception of a minimal biological marker is necessary to be a target for identifying a model for learning or for be a target for instruction. Thanks to Sally Haslanger for making this more salient to me.

8 I am inclined to think that the possibility of the two genders merging into a single gender and one or two genders becoming extinct for most practical purposes amounts to similar scenarios.

9 Ursula Le Guin’s *Left Hand of Darkness* (2012) describes a society in which humans are not thought of or classified according to gender, but are effectively genderless androgynes most of the time. Nor is their sex determinate. During their fertile periods (kemmer) these individuals adopt either the female or the male sex. In the real world, however, it is quite unclear what a genderless system or a system in which gender is eliminated would be like.

10 Depending on the details of the case these individuals would, according to the historical account, belong to both genders or neither gender (and thereby also stand as a possible model for a new gender).

11 Although it is not clear whether it is cultural *group* selection that he has in mind or if it is also an advantage to the individual trait bearer’s reproductive fitness.

12 I would like to thank one of this journal’s reviewers for prompting more general reflection on this strategy for solving the representation problem, which indeed refocused the argument set out in this paper.

13 See Shelby (2005) for a developed statement about social-cultural links of solidarity in notions of race.

14 I thank Sune Hannibal Holm for making this provocative proposal.

15 On this option, it is also open to us to restate womanhood in a case of affirmative action as indexed to a particular culture (i.e. Zuni culture or Swedish culture)—but it should be acknowledged that such indexing is typically not thought to be necessary when making claims about affirmative action.
